# Motivations for urban front gardening: A quantitative analysis

**DOI:** 10.1016/j.landurbplan.2023.104835

**Published:** 2023-10

**Authors:** Niamh Murtagh, Rachael Frost

**Affiliations:** a The Bartlett School of Sustainable Construction, University College London (UCL), 1-19 Torrington Place, London WC1E 7HB, United Kingdom; b Department of Primary Care and Public Health, University College London (UCL), UCL Royal Free Campus, Rowland Hill Street, London NW3 2PF, United Kingdom

**Keywords:** Ecosystem services, Motivation, Nature-based solutions, Self-determination theory, Urban front gardens

## Abstract

Private gardens in urban settings offer multiple benefits for the environment and society. In addition to benefits to people’s health and well-being, planting in front gardens in particular can mitigate local flooding and urban heat islands. To encourage more front garden planting, greater understanding of householders’ motivations for front gardening is needed. Addressing research gaps on gardening for reasons other than food production and on motivations for gardening in front gardens, a large-scale online survey (n = 1,000) was conducted with urban/suburban dwellers in England. Exploratory factor analysis identified three factors of motivation: enjoyment, meaning and benefit (intrinsic), creating something beautiful (aesthetic) and functional outcomes (utilitarian). A multiple regression model incorporating the three factors and sociodemographic variables explained 11% of variance of time spent front gardening, with intrinsic motivations the strongest predictor. Intrinsic motivations were stronger for women than for men. The study provides a quantitative categorisation of motivational factors as a basis for comparative research and design of interventions and policy to increase front gardening.

## Highlights

• Private urban gardens, especially front gardens, offer valuable eco-system services.
• Understanding motivations for front gardening can help to maintain vegetation levels.
• We conducted factor and regression analyses on data from a large UK survey (n = 1,000)
• Enjoyment and benefit were the strongest motivations for time spent front gardening.
• Motivations for beauty and functionality were also influential.

## 1 Introduction

The effects of climate change and biodiversity loss can be mitigated by green infrastructure in cities (Cameron & Blanusa, 2016). The environmental and social benefits of vegetation, termed ecosystem services (Cameron & Blanusa, 2016) or Nature-based Solutions (Puskás, Abunnasr, & Naalbandian, 2021), include many of particular importance for coping with global warming such as carbon sequestration, rainwater retention, cooling and improving human health and well-being. Vegetation and soil provide habitats for diverse fauna, from microscopic organisms and fungi, to invertebrates, birds and mammals (IPBES, 2019). Extensive evidence has shown the human health benefits of exposure to nature, particularly the benefits to well-being and mental health (de Bell et al., 2020). As will now be discussed, urban gardens offer valuable space for planting, but this has been diminishing, and while research has offered insights into motivations around gardening, many previous studies have been small-scale and have, by and large, overlooked front gardens.

### 1.1 Urban gardens

Around the world, private gardens represent significant space with the potential to provide ecosystem services. Across the UK, 88% of homes in the UK have garden space and the combined acreage is estimated as 7,289 km^2^ (ONS, 2020), that is, larger than twice the size of the Peak District and Snowdonia National Parks combined. In South Africa, an estimated three quarters of vegetated space in urban settings is on private land (Shackleton et al., 2018). In terms of biodiversity support, urban gardens provide so-called ‘green corridors’ which are vital to reduce the impact of loss of space by allowing fauna to move between habitats that remain (Guneroglu, Acar, Dihkan, Karsli, & Guneroglu, 2013).

There is evidence that these valuable green spaces are being lost (Bonham et al., 2019, Jaganmohan et al., 2012, Smith et al., 2011). Of particular concern is the loss of front garden vegetation, that is, greenery between the façade of a property and the public street. Research in Edinburgh showed that the impact of paved front gardens is already increasing flooding incidence (Kelly, 2018) and there is evidence from Greece of the importance of planting to reduce summer air temperatures (Tsilini, Papantoniou, Kolokotsa, & Maria, 2015). The loss of urban garden space has been reported globally, from Germany (Wellmann, Schug, Haase, Pflugmacher, & van der Linden, 2020), to India (Balooni, Gangopadhyay, & Kumar, 2014) to Ecuador (Finerman & Sackett, 2003) for reasons including both formal planning regulations and informal development. In order to encourage urban dwellers to maintain greenery, we need to understand motivations for gardening, especially for gardening in front gardens. While there may be different motivations at play driving removal of vegetation (e.g. paving over what was previously planted) versus maintaining or increasing planting, our study takes the latter as an initial focus to develop deeper understanding.

### 1.2 Motivations for gardening

Early work on gardens in suburban housing estates in England noted that functional motivation prevailed, with back gardens perceived as an extension of indoor space, and used for sitting or eating outside, children playing, clothes drying and gardening (Cook, 1968). In California, gardens have been categorised as: living gardens, which aligned with Cook’s (1968) functional motivations; well-tempered gardens, in which householders desired a controlled, neat appearance with non-natural decorative features; and expressionist gardens, in which householders demonstrated varied motivations (Grampp, 1990). Regarding front gardens, Cook (1968) argued that front gardens had been kept for display until the late 1940 s but were shrinking in size. In the US, the condition of front gardens and lawns in particular has been proposed to be motivated in part by community commitment (Harris & Brown, 1996). A focus on front gardens has been relatively rare in previous work but this gap has been addressed in part by a recent, multi-disciplinary field study in an economically-deprived setting in northern England (Chalmin-Pui, Roe, et al., 2021). The findings of reduced stress levels, measured via cortisol, and of improved subjective well-being, measured on an established self-report instrument, provide important and robust evidence supporting the importance of front gardens in particular to individual health. Chalmin-Pui et al. (2019) outlined the differences between front and back gardens which include functional or utilitarian differences, and emphasised the semi-private nature of front gardens, which act as a buffer between private and public spaces and can offer public as well as private benefits.

Much recent research has focused on community gardening, allotments and urban agriculture (e.g. Kirkpatrick and Davison, 2018, Zasada et al., 2020). Indeed, the terms ‘urban gardening’ and ‘urban agriculture’ are frequently used interchangeably (Ruggeri, Mazzocchi, & Corsi, 2016). Motivations for gardening are likely to correspond strongly with the anticipated benefits and setting (Ruggeri et al., 2016). Many studies do not distinguish between gardening for food production and other forms of gardening, with relatively few investigating private gardens. Within the psychological and wider literature, there has been discussion of gardens as therapeutic spaces (Murroni et al., 2021), as bringing health and wellbeing benefits to older people (Wang & MacMillan, 2013) and to children (Skelton, Lowe, Zaltz, & Benjamin-Neelon, 2020). There has been passing reference to gardens as factors contributing to values and identity (Francis, 1990), as important for escapism, identity and ownership (Gross & Lane, 2007), and the relationship between an environmental gardening identity and ecological gardening practices has been examined (Kiesling & Manning, 2010). Qualitative work in New Zealand suggested motivations relating to physical and mental health, expression of ownership and identity, socialising, nature connection and food production (Freeman, Dickinson, Porter, & van Heezik, 2012). A large-scale (n = 6015) UK study asked a single, open response item ‘Why do you garden?’ and provided descriptive statistics: more than half of respondents indicated pleasure and enjoyment as a reason (Chalmin-Pui, Griffiths, Roe, Heaton, & Cameron, 2021). Other common responses were sensory reasons, health benefits, seeing plants grow, expression and self-identity.

An early attempt at systematic classification of motivations for gardening was an exploratory study with a convenience sample in the US (Clayton, 2007) which listed benefits of gardening as including aesthetic, mental and physical health benefits. A recent small-scale qualitative study proposed themes of self-identity, community, fulfilment and health, based on UK gardeners involved in a national competition (Chalmin-Pui, Griffiths, Roe, & Cameron, 2021). A focus group study, also in the UK, conducted as initial exploratory research on which to base the current study, noted effort and reward, connecting with outdoor spaces, social benefits, and the need for gardening knowledge and self-efficacy, for a mixed UK sample of gardeners and non-gardeners (2023, insert ref after review). In Milan, Italy, analysis of 60 survey responses determined two underlying dimensions of motivation: physical and psychological well-being, including learning new skills and benefits for the local environment (Ruggeri et al., 2016). In Muscat, Oman, a survey of 125 respondents found motivations to include aesthetic benefits, shading, joy, food, exercise and other benefits to the environment (Al-Mayahi, Al-Ismaily, Gibreel, Kacimov, & Al-Maktoumi, 2019).

While there are rich qualitative findings and mainly small-scale quantitative evidence relating to motivations for front gardening, what is not clear is how such various motivations relate to each other: are the motivations for exercise and for sensory benefits distinct from or part of physical and psychological motivations, for example? What is missing is large scale, replicable analysis that enables identification of the smallest justifiable set of motivational factors, and inferential statistics to test the relationship of these motivational factors with the outcomes of interest. This will facilitate further research by clustering similar motivations together and distinguishing between different clusters (Tabachnick & Fidell, 2001) and reducing the unwieldy list of motivations to a small set while retaining as much original information as possible (Field, 2005). Such an approach can be replicated across contexts, countries and timepoints, enabling comparative analysis, and understanding these clusters allows for improved design of future public health and urban environmental interventions by ensuring they are based on data gathered from a broad enough sample to target a large number of the intended population.

Addressing the research gaps on the structure of motivations for gardening in private, urban gardens, and focusing on front gardens in particular due to the potential importance of their ecosystem services for nature-based solutions, the current study presents the first application of exploratory factor and inferential statistical analyses to a large-scale survey of motivations to garden in front gardens. The research questions were: What motivates front gardening among urban/suburban residents in England? How may such motivations be structured?

## 2 Methodology and method

The survey was designed by the authors and conducted online in May 2021. To extend previous work which has frequently focused on older gardeners (Nicholas, Giang, & Yap, 2019), we chose to focus on working age adults. A market research organisation was used to recruit adults between 20 and 65 years of age, with quotas of half under 43 years (mid-point), an ethnicity mix to include approximately 6% Asian or British Asian and 3% Black or Black British to reflect the UK general population (ONS, 2011), a gender ratio of 65:35% or better balance, and a ratio of homeowners to tenants of approximately 65:35% to reflect the national rate (ONS, 2012). Participants were screened to ensure they had ground floor front garden space between their home and the street at least large enough for three recycling bins.

The survey collected data on the following additional socio-demographics: age and number of children (integer responses); gender (woman/man/other); employment status, income and level of education (categorical responses) and approximate front garden size. Employment status ranged from full-time employment outside the home to retired and was tentatively interpreted as an indicator of time available. Dependent variables measured were: time in minutes spent front gardening in an average week in summer (TIME) and percentage of greenery in front garden (GREEN), based on a categorical variable which used a text description alongside a numeric percentage to aid ease of interpretation by participants:

Approximately how much of your front garden has got plants, grass, flower bed or shrubbery?

0% (none at all); 20% (a small amount); 40% (nearly half); 60% (more than half); 80% (three quarters or more); 100% (it’s all covered in plants, grass, flower bed or shrubs).

We also asked ‘Do you have a disability which limits your ability to do gardening?’ and analysed this group separately (n = 124) to assess if a disability constraint might affect motivations.

Based on an initial qualitative study documented in depth elsewhere (reference after review), we generated a set of 17 items to describe possible motivations to engage in front gardening. Noting a gap related to awareness of wider environmental benefits of front gardens beyond supporting wildlife, a further three items relating to environment were added to the list, specifically on reducing climate change, local flooding and keeping homes cooler in hot weather. The full list of items is presented in Table 2.

The data were inspected for outliers. Sixteen cases were excluded due to poor data quality. We conducted Exploratory Factor Analysis (EFA) on the 20 items relating to motivation to do front gardening; linear regressions on the dependent variables described above (TIME, GREEN) of the EFA factors and sociodemographics; and finally the same linear regressions split by gender to explore gender differences. This was of interest because household tasks are commonly gendered in practice, with men more likely to do outdoor chores (Quadin & Doan, 2018). Questions on motivations for front gardening were only asked of participants who indicated TIME greater than zero, and EFA and regressions were conducted for these participants (n = 694).

The survey was approved by the lead author’s Departmental Ethics Committee. The survey was fully anonymous and informed consent was gained before the start of data collection, with the right to withdraw at any point up until submission of the completed questionnaire.

## 3 Results

Table 1 provides summary demographic characteristics of the survey respondents, demonstrating that the intended sampling targets were achieved. Table 2 presents correlations of socio-demographic variables.

**Table 1:** Respondent Sociodemographics (n = 1,000).

Variable	Description
Age	Mean 45.47, std. dev. 11.8
Gender	Woman 57.8% Man 41.9% Other 0.3%
Ethnicity	White British 85.8% Asian or Asian British 7.0% Black or Black British 3.2% Other 4.0%
Home ownership	Homeowner 67.1% Tenant 32.4% Other 0.5%
Income	Less than £3,001 monthly net income 59.5% More than £3,000 monthly net income 34.9% Not given 5.6%
Employment status^a^	Full-time employed mainly outside the home 43.4% Full-time employed mainly at home 13.2% Part-time employed 15.0% Full-time homemaker or carer 11.9% Student 1.1% Seeking employment 3.4% Furloughed^b^ 0.2% Retired 11.4% Other 0.4%

Notes: *a* Categories listed in reverse order of assumed time available (see discussion in this section and Section4 Discussion). *b* ‘Furloughed’ indicated individuals not working because of pandemic lockdowns but receiving pay.

**Table 2:** Correlations (n = 1,000).

	Age	Education	Income	Employment status	Children
Age	–				
Education	-0.055	–			
Income	0.107^**^	0.279^**^	–		
Employment status	0.287^***^	-0.159^***^	-0.187^***^	–	
Children	-0.230^***^	-0.039	0.001	-0.094^**^	–
Average front garden size	0.155^***^	0.105^**^	0.183^***^	-0.021	0.035

Note: ** *p* <.01; *** *p* <.001.

The correlations suggested that, within the 20 to 65 year age range of the sample, the older participants were likely to earn more and to have a larger garden. For employment status, the lower values on the scale represent full- and part-time employment and, as expected, the measure correlates negatively with education and income.

Exploratory Factor Analysis (EFA) was conducted on the 20 items relating to motivations to do front gardening. Inspection of the correlation matrix showed that almost all were correlated, with no correlation coefficients over 0.8, indicating the data set was suitable for EFA. This was confirmed by a KMO measure of sample adequacy of 0.96 which is considered excellent (Hutcheson & Sofroniou, 1999) and a significant outcome on Bartlett’s test of sphericity. EFA was conducted using maximum likelihood, as most appropriate for a randomly selected sample, and direct oblimin rotation, assuming some correlation between items (Field, 2005). The EFA showed 3 factors with eigenvalues of greater than 1, cumulatively accounting for 60.13% of variance, and 48.48%, 7.82% and 3.83% for Factors I, II and III respectively. Table 3 presents the pattern matrix.

**Table 3:** Pattern matrix, with maximum likelihood extraction and direct oblimin rotation (n = 694).

	Factor		
	I	II	III
Because I like caring for plants	**0.87**	-0.02	-0.06
Because of the enjoyment of physical activity	**0.85**	0.04	-0.01
Because of the satisfaction of seeing things grow	**0.84**	-0.22	-0.13
Because of the enjoyment of fresh air, sun and nature	**0.84**	-0.21	-0.11
Because of the enjoyment of the sounds, smells and sensations of gardening	**0.83**	-0.04	-0.05
Because of the mental health benefits such as reducing stress or relaxing	**0.83**	0.08	0.01
For the enjoyment of completing projects in my front garden	**0.80**	-0.10	0.03
Because I enjoy pottering	**0.78**	0.01	-0.00
Because it allows me to focus on just what I’m doing at that moment	**0.74**	0.03	0.11
To help local wildlife like bees	**0.72**	-0.02	-0.01
To do my bit in reducing climate change	**0.62**	0.15	0.22
Because of the physical health benefits eg weight control or building strength	**0.45**	0.22	0.33
Because it’s a sociable activity	**0.44**	0.12	0.43
To create a pleasant space for socialising	**0.42**	0.10	0.42
To make the front look good for me and my household	0.41	**-0.60**	0.04
To make the front look good for passers-by or neighbours	0.18	**-0.60**	0.46
Because my neighbours would expect me to	-0.14	-0.10	**0.63**
To help avoid or reduce local flooding	0.31	0.29	**0.48**
To keep my home cooler in hot weather	0.37	0.35	**0.48**
Because it adds to the value of my home	0.33	-0.09	**0.42**

Note: figures in bold indicate the factor on which an item loads.

We interpreted these factors as:

• Intrinsic or internalised motivations (Factor I, 14 items) which relate to enjoyment and personal benefit, as well as gardening for biodiversity and climate change
• Aesthetic motivations (Factor II, 2 items) which refer to creating something beautiful
• Utilitarian motivations (Factor III, 4 items) which relate to a functional view - what can the front garden usefully do in terms of local environmental impact and value, as well as local norms.

In Factor I, gardening for biodiversity and climate change may represent internalised motivations, that is, motivations that have become important to the individual. We noted that two items that loaded on Factor 1 also loaded to a similar level on Factor 3 (a social activity; a pleasant space for socialising), suggesting that these items may have both internalised and utilitarian motivations. The interpretation of Factors I, II and III is supported by the fact that the two items loading highest on Factor II also loaded on another factor each, albeit with a lower weighting: making the front of the home look good for one’s own household also loaded on Intrinsic/internalised motivations; making the front look for others also loaded on Utilitarian motivations. To assess the reliability of the 3 factors as scales, calculation of Cronbach alpha gave 0.95, 0.71 and 0.72 for Factors I, II and II respectively, indicating excellent to acceptable reliability (Field, 2005).

The three motivation scales were then included in linear regressions to assess if they added explanation of variance over and above sociodemographic factors. Two dependent variables (DVs) were evaluated: Average Time spent Front Gardening (TIME) and Percentage of front garden that is Green (GREEN). GREEN had a normal distribution. TIME was negatively skewed and was transformed by taking the square root. There were no indications of multicollinearity: variance inflation factors averaged less than 1.5. Table 4 presents the results.

**Table 4:** Results of linear regressions.

	TIME			GREEN		
	B [95% Confidence Int. lower, upper]	Beta	Sig.	B [95% Confidence Int. lower, upper]	Beta	Sig.
Age	**-0.08 [-0.13, -0.03]**	**-0.13**	**0.00**	-0.00 [-0.01, 0.01]	-0.02	0.64
Education	-0.10 [-0.46, 0.25]	-0.02	0.57	**0.07 [ 0.00, 0.14]**	**0.08**	**0.05**
Income	-0.03 [-0.37, 0.32]	-0.01	0.90	0.03, [-0.04, 0.10]	0.04	0.35
Employment status	**0.35 [ 0.11, 0.60]**	**0.11**	**0.01**	**0.05 [ 0.00, 0.10]**	**0.08**	**0.04**
Children	**0.51 [ 0.01, 1.00]**	**0.08**	**0.04**	-0.09 [-0.18, 0.01]	-0.07	0.09
Front garden size	0.01 [-0.00, 0.12]	0.07	0.09	**0.00 [ 0.00, 0.00]**	**0.12**	**0.00**
Intrinsic motivation	**1.95 [1.35, 2.54]**	**0.35**	**0.00**	**0.13 [ 0.01, 0.25]**	**0.12**	**0.03**
Aesthetic motivation	-0.36 [-0.84, 0.12]	-0.06	0.15	**0.14 [ 0.05, 0.24]**	**0.13**	**0.00**
Utilitarian motivation	-0.40 [-0.94, 0.13]	-0.07	0.14	-0.09 [-0.20, 0.01]	-0.09	0.08
		Adj *R^2^* = 0.11, *F*(9,684) = 10.21, *p* =.00			Adj *R^2^* = 0.06, *F*(9,684) = 6.27, *p* =.00	

Notes: n = 694. TIME = Average time spent front gardening; GREEN = Percent green. Beta = standardised coefficients. Significance at *p* <.05 in **bold**. Adj *R^2^* = percentage variance explained by the model.

The model for TIME explained approximately 11% of variance. Three sociodemographic variables contributed: age, employment status and number of children. Interestingly, age was negatively associated with time spent front gardening. Employment status was interpreted as a proxy for available time at home, with full-time work out of or at home representing lower values as indicated by their order in Table 1, so the significant relationship here may indicate time available. Income was not related to time spent front gardening. The strongest determinant was intrinsic motivation and neither aesthetic nor utilitarian motivations were significantly related to front gardening time.

The percentage of greenery in the front garden offered a different outcome of relevance. Level of education explained a small amount of variance, as did employment status/time available. Front garden size was significant, indicating perhaps that more greenery is more practical in a larger space. Intrinsic motivation was significantly related to the percentage of greenery, and so too was aesthetic motivation: wanting the front to look good was a significant factor. Utilitarian motivations did not significantly contribute to percentage of greenery.

For the participants indicating that a disability affected their time spent front gardening (n = 124), we found some differences from the results in Table 4. For time spent front gardening (TIME), Intrinsic motivations (beta = 0.39, *p* =.02) and the number of children (beta = 0.35, *p* =.00) were significant as they were for the majority of participants but neither age nor employment status were significant factors, while Utilitarian motivations were a negative factor (beta = -0.30, *p* =.03). For percentage of greenery in the front garden (GREEN), only age (beta = 0.25, *p* =.02) and Aesthetic motivations (beta = 0.26, *p* =.04) were significant.

We then split our sample by gender (omitting two cases of Non-binary/Other gender) and reran the regressions to explore any differences (see Table 5).

**Table 5:** Results of linear regressions by gender.

	Female				Male			
	**TIME**		**GREEN**		**TIME**		**GREEN**	
	Beta	Sig.	Beta	Sig.	Beta	Sig.	Beta	Sig
Age	**-0.19**	**0.00**	0.03	0.67	-0.08	0.27	-0.13	0.08
Education	-0.07	0.13	**0.13**	**0.01**	0.08	0.19	0.03	0.67
Income	0.07	0.17	0.05	0.38	**-0.15**	**0.02**	-0.01	0.89
Employment status	0.09	0.10	0.06	0.31	**0.13**	**0.05**	0.12	0.06
Children	**0.11**	**0.03**	-0.03	0.54	0.01	0.82	-0.09	0.13
Front garden size	**0.10**	**0.04**	**0.11**	**0.03**	0.05	0.45	**0.14**	**0.02**
Intrinsic motivation	**0.39**	**0.00**	**0.15**	**0.03**	**0.24**	**0.01**	0.04	0.64
Aesthetic motivation	-0.09	0.09	**0.14**	**0.02**	0.01	0.93	**0.16**	**0.02**
Utilitarian motivation	-0.11	0.06	-0.07	0.27	0.01	0.88	-0.11	0.22
	Adj *R^2^* = 0.15 *F*(9,378) = 8.75, *p* =.00		Adj *R^2^* = 0.09 *F*(9,378) = 5.21, *p* =.00		Adj *R^2^* = 0.08 *F*(9,295) = 3.84, *p* =.00		Adj *R^2^* = 0.03 *F*(9,295) = 2.1, *p* =.03	

Notes: n = 693. Beta = standardised coefficients. Significance at *p* <.05 in **bold**.

The gender analysis showed some interesting differences. For time spent front gardening, age was only significant for women, suggesting that younger women were more likely to spend time front gardening. Women with more children and a larger front garden were also more likely to garden there. Interestingly, there was a negative relationship between income and time spent front gardening for men: men with higher incomes were less likely to work on the front garden. Employment status made a contribution for men only, implying that men with more time at home may do more front gardening. For both women and men, the strongest determinant of time spent front gardening was intrinsic motivation.

In terms of percentage of greenery, women’s education was a positive factor, and for both women and men, a larger front garden increased the reported proportion of greenery. For women only, intrinsic motivation made a difference. For both women and men, the aesthetics of the front of the home was a factor contributing to more greenery.

## 4 Discussion

The study aimed to identify a small, structured set of motivations for gardening in front garden, based on a large scale sample and replicable methodology. Using a 20-item measure of motivations based on earlier focus group findings (insert ref after review) and wider literature, an exploratory factor analysis (EFA) was conducted on a survey of 1,000 respondents across England and found a structure of three motivational factors: intrinsic motivations, aesthetic motivations, and utilitarian motivations, accounting for 60% of variance.

The dominant factor comprised mainly intrinsic motivations, aligning with the theoretical predictions of Self-Determination Theory (Ryan & Deci, 2017) that intrinsic motivations are the strongest determinants of behaviour. These included embodied physical enjoyment (pleasure in physical activity, fresh air and sun, sensations), psychological benefits (reduction in stress, engagement and focus on activities) and goal-fulfilment (seeing things grow, completing projects). Unexpectedly, Factor I (Intrinsic motivations) included two items on the natural environment. One related to biodiversity. Earlier research noted a moral responsibility to care for wildlife (Goddard, Dougill, & Benton, 2013) which implies an internalisation of the importance of gardens for wildlife. The second related to climate change more generally. Public awareness of the climate crisis has grown over recent years (Whitmarsh, 2020) and it is feasible that gardening for climate change is also becoming internalised as a motivation. Social desirability may also have played a role, whereby people feel that they *should* indicate that they are taking action to mitigate climate change. The framing of the items (To help local wildlife like bees; To do my bit in reducing climate change) could equally have triggered altruistic tendencies, and there is strong evidence of the relationship between altruism, pro-environmental behaviours and intrinsic motivation (Ali et al., 2020, Cecere et al., 2014). The findings suggest that biodiversity and climate change motivations can be included as items in in measures of intrinsic motivations for gardening.

It is noteworthy that the intrinsic motivations of Factor I align with the notion of ‘doing’ gardening while the aesthetics of the front garden, identified as Factor II in the EFA, align with ‘having a garden’. With a few exceptions such as Chalmin-Pui, Griffiths, Roe, and Cameron (2021), these alternative perspectives – of gardening as an activity versus a (nice) garden as an end goal – are usually confounded in research, but merit separate consideration. Different psychological processes may underlie each (such as social identity in the former and aesthetic preferences in the latter) and more detailed understanding could point to different behavioural intervention techniques to engage more people in front gardening. Factor II is a valuable reminder that the appearance of the garden matters, also noted by Chalmin-Pui and colleagues (Chalmin-Pui, Griffiths, Roe, & Cameron, 2021). This is particularly true for the front garden which is usually open to view by passers-by and visitors, and its aesthetic is likely to be strongly influenced by social norms (Nassauer, 1997).

We titled Factor III as Utilitarian motivations which encompassed aspects of providing protection from the changing climate, real estate value, as well as social norm pressure. Such motivations are closer to extrinsic or controlled motivations on the spectrum posited in Self-Determination Theory (Ryan & Deci, 2017) but it is somewhat difficult to plot utilitarian motives on this spectrum: an individual may feel that increasing their house value is important to them (an identified motivation) or may feel that they should do so (an introjected motivation). For this reason, Factor III has not been named as extrinsic motivations. The finding of social norm pressure in Factor III is likely to apply particularly to front gardens. It speaks to an understanding by householders that there are social or cultural expectations around how their front garden looks and what it should do. In our earlier qualitative study, participants prioritised ensuring the front garden looked neat and tidy, and acknowledged that this was also a pressure from neighbours (insert ref after review). Although the regressions showed Factor III to be non-significantly related to time spent gardening or percentage of greenery compared to Factors I and II, this may still be an important additional lever to explore in interventions to change front gardening behaviour. In particular, its negative relationship with time spent front gardening for participants indicating a disability suggested that functional motivations could work differently for different groups. If informational campaigns can raise awareness of what ecosystem services can be provided by front gardens, with campaigns highlighting local problems with flash flooding or excessive heat for example, this could contribute to social expectations on how front gardens should help to protect the neighbourhood. Our findings suggest that this could increase motivations to increase planting in front gardens.

The negative relationship between age and time spent front gardening was initially surprising, with previous evidence suggesting that time spent gardening increases with age (de Bell et al., 2020). The maturity of the garden may play a role: the finding could depict a pathway of buying a house, having children and spending more time on front gardening in earlier adult years, with the garden developing into an acceptable form and needing less work in later years. This is consistent with the positive relationship between number of children at home and time spent front gardening. An alternative explanation could be that the increasing caring responsibilities of middle-aged people, who may be looking after older relatives as well as children or grandchildren, combined with the demands of a job and possible greater seniority, leave little time for gardening. This explanation is supported by the relationship between age and time spent front gardening which is negative and significant for women, who tend to carry the major burden of caring responsibilities (Andersen, Toubøl, Kirkegaard, & Bang Carlsen, 2021), but non-significant for men. The lack of relationship between age and percent green implies that gardens may not become greener as householders get older. Interestingly, we did not find a relationship between income and either time spent front gardening or percent green, indicating that cost was not an important factor for green front gardens. This was consistent with findings in our earlier qualitative study, where cost related more to a desire to avoid wasted expense from choosing plants that subsequently died than any substantive concerns about general costs of gardening (insert ref after review).

For participants who indicated a disability, the findings that the three motivations showed significant relationships with the dependent variables provides additional support for the salience of the factors for different groups. The slight differences in factor profile between those indicating a disability and the remainder of the participant group suggests that different motivational patterns may apply for different groups. Future research could aim to evaluate the three factors with specific social groups in order to tailor campaigns and interventions appropriately.

Analysis by gender revealed some intriguing differences. Intrinsic motivations were stronger for women than for men. Are women more aware of the intrinsic benefits or value them more? Is there a tendency for men to conduct front gardening more as a chore than an intrinsic pleasure, with a more goal-oriented approach as a stronger motivation? Grampp (1990) had noted that the ‘well-tempered’, controlled, neat garden represented the garden as a project, and was more likely to be male-worked. The negative relationship with income, which held only for men, could support this interpretation: a man earning more may be more likely to pay someone else to look after the front garden. Based on our use of employment status as a proxy for time available, men, but not women, were more likely to spend time front gardening if they had more time available. The evidence for women continuing to carry the majority of the domestic burden (Cerrato & Cifre, 2018) would suggest that women folded gardening tasks into their workload. Equally the intrinsic enjoyment may have more importance for women. These speculations point to questions for future research on gendered motivations for gardening.

Limitations of the study require acknowledgement. The survey was conducted in England and may reflect country-specific cultural norms, habits, geography and climate. It was carried out in May 2021, in the context of Covid-19 pandemic control measures. There is evidence of an increased interest in gardening during this time (Lin et al., 2021), and front gardens took on particular importance, as outdoor space where people could socialise at a distance (Gordon-Rawlings & Russo, 2023). Future research can trace if motivations have changed since this exceptional period.

Our use of employment status as a proxy for time was a possibly weak solution to the problem of measuring ‘time available’, particularly in the context of increased working from home during the Covid-19 pandemic. From our earlier qualitative work (reference after review), the perception of ‘time available’ was an important factor for participants. However, this is a highly subjective concept, confounded with motivation, for example, I am more likely to make time for what is important to me but less likely to find time for activities I do not wish to do. Previous research has shown that empirical and perceived time are not identical, and that perceptions of time can be influenced by factors such as enjoyment of an activity, frequency of engagement, meaning and value associated with an activity, individual differences including gender, and between perception of time and its representation to others (Hornik, 1984). Distinguishing between real constraints versus psychological framing, or perception, of time available, presents a challenge, particularly to research on motivations for front gardening. A possible way forward is for future research to use more detailed time measures such as those in the established Time Use Survey (Anderson, 2016). Alternative approaches such as diary studies could provide more accurate data on time spent front gardening. Our focus on motivations necessarily sidelined broader contextual factors such as the garden setting, types of planting or participant knowledge and skills. For future work, our findings suggest that the role of gender is important to examine in more depth and that there is a need for more work on the aesthetics and cultural norms of front gardens.

## 5 Conclusions

The study offered a first, large-scale, quantitative approach to identifying motivations for front gardening and green front gardens on the basis of exploratory factor analysis and inferential statistics. Aligning with Self-Determination Theory, an established theoretical framework on motivation, the findings of three motivational factors – intrinsic, aesthetic, and utilitarian motivations – contributes to theoretical understanding of front gardening. The method is replicable across other contexts and could be used for comparative analysis between different geographic areas, for example. The three-factor motivational structure can be used as a basis for design of interventions in line with the Behaviour Change Wheel approach to behaviour change (Michie, Van Stralen, & West, 2011) offering implications for interventions and policy approaches to encourage more front garden planting and address the low engagement with Nature-based Solutions found in earlier research (Dorst et al., 2022).

Policy and interventions should target the intrinsic motivations first, as this is the dominant factor. While intrinsic motivations can drive behaviour relatively independently of the external context, raising the salience of the pleasure of being outside, of being physically active and of producing something beautiful may resonate with many. In addition, highlighting well-being and physical health benefits will encourage some individuals, and connects with public health messages on physical activity. There is scope to target the look of front gardens: this will require developing and supporting social norms around planting in front gardens. Expanding resourcing on campaigns such as Britain in Bloom, an annual gardening competition (RHS, 2023), would target this motivational factor.

Building knowledge of the benefits for flood and overheating reduction, and of the potential for green front gardens and trees to add value to the home (Chen and Jim, 2010, Conway et al., 2010, Nesticò et al., (2020, 2020//).), could leverage utilitarian motivations. Use of historical photos of local streets with far more greenery could be an approach to starting to change expectations in neighbourhoods. Income is not a significant determinant and although time may be a factor, there is scope to emphasise the potential to increase greenery even in small gardens. There may be better times in the lifecycle to encourage people to add more greenery, for example, targeting younger, working-age adults and families with young children where both parents do not work full-time outside the home. Targeting relevant motivations for green front gardens will enable better utilisation of valuable resources that can help to mitigate the extent and impact of climate change in urban spaces.

## CRediT authorship contribution statement

**Niamh Murtagh:** Conceptualization, Funding acquisition, Methodology, Investigation, Writing – original draft. **Rachael Frost:** Conceptualization, Funding acquisition, Methodology, Investigation, Writing – review & editing.

## Declaration of Competing Interest

The authors declare that they have no known competing financial interests or personal relationships that could have appeared to influence the work reported in this paper.

## Data Availability

Data will be made available on request.
